# Influence of sediment and stream transport on detecting a source of environmental DNA

**DOI:** 10.1371/journal.pone.0244086

**Published:** 2020-12-28

**Authors:** Meredith B. Nevers, Kasia Przybyla-Kelly, Dawn Shively, Charles C. Morris, Joshua Dickey, Murulee N. Byappanahalli

**Affiliations:** 1 Great Lakes Science Center, U.S. Geological Survey, Chesterton, Indiana, United States of America; 2 Department of Civil & Environmental Engineering, Michigan State University, East Lansing, Michigan, United States of America; 3 Indiana Dunes National Park, National Park Service, Porter, Indiana, United States of America; University of Hyogo, JAPAN

## Abstract

Environmental DNA (eDNA) can be used for early detection, population estimations, and assessment of potential spread of invasive species, but questions remain about factors that influence eDNA detection results. Efforts are being made to understand how physical, chemical, and biological factors—settling, resuspension, dispersion, eDNA stability/decay—influence eDNA estimations and potentially population abundance. In a series of field and controlled mesocosm experiments, we examined the detection and accumulation of eDNA in sediment and water and the transport of eDNA in a small stream in the Lake Michigan watershed, using the invasive round goby fish (*Neogobius melanostomus*) as a DNA source. Experiment 1: caged fish (average n = 44) were placed in a stream devoid of round goby; water was collected over 24 hours along 120-m of stream, including a simultaneous sampling event at 7 distances from DNA source; stream monitoring continued for 24 hours after fish were removed. Experiment 2: round goby were placed in laboratory tanks; water and sediment were collected over 14 days and for another 150 days post-fish removal to calculate eDNA shedding and decay rates for water and sediment. For samples from both experiments, DNA was extracted, and qPCR targeted a cytochrome oxidase I gene (COI) fragment specific to round goby. Results indicated that eDNA accumulated and decayed more slowly in sediment than water. In the stream, DNA shedding was markedly lower than calculated in the laboratory, but models indicate eDNA could potentially travel long distances (up to 50 km) under certain circumstances. Collectively, these findings show that the interactive effects of ambient conditions (e.g., eDNA stability and decay, hydrology, settling-resuspension) are important to consider when developing comprehensive models. Results of this study can help resource managers target representative sites downstream of potential invasion sites, thereby maximizing resource use.

## Introduction

Invasive species, such as round goby *(Neogobius melanostomus*), quagga and zebra mussels (*Dreissena bugensis* and *D*. *polymorpha)*, and the carp species (bighead (*Hypophthalmichthys nobilis)*, silver carp (H. *molitrix)*, black carp (*Mylopharyngodon piceus)*, and grass carp (*Ctenopharyngodon idella*), have greatly impacted the Great Lakes and/or their associated waterways by disrupting the natural ecosystems and overall function [1]. Thus, early detection of invasive species is critical, to allow resource managers time to develop and implement strategies for eradication and/or control [2] and to minimize spread to new areas. Too often, invasive species invade a system and secure a foothold before their presence is detected, making control efforts decidedly more difficult [3]. The long-term impacts of invasion can be catastrophic to native biodiversity, with the alteration of food web structures [1, 4, 5] and the potential for massive economic impacts [6]. In the Laurentian Great Lakes of North America, the cascading impact of the invasive quagga and zebra mussels over several decades [7] has motivated resource managers (federal, state, tribes, local) and researchers to increase efforts to detect new invasive species early, by using advanced technologies such as environmental DNA [3, 8, 9] and an array of emerging technological innovations (e.g., drones, sensors, satellite imagery) [10].

The use of environmental DNA (eDNA) is expanding rapidly in ecological studies (see [11]). The possibilities for detecting and identifying target organism presence without physically sighting or capturing specimens has expanded applications of this technology from ancient anthropology [12] to deep-sea fisheries [13], to pollinator behavior [14]. With increase in demand for eDNA tools, including early detection or warning to halt the spread of invasive species, scientists are diligently working to improve methods of detection and results interpretation in the laboratory and in the field to define more clearly what it means when eDNA is detected in a natural waterway [10].

Environmental DNA originates from multiple biological processes, including cell or tissue shedding, defecation, egg deposition, and natural organism decay [15–17]; once it is released in aquatic systems, viability and detectability (fate) are affected by biological, chemical, and physical processes that may accelerate/prolong decay [18]. The rate of release for a given species may be affected by organism life (physiological) stage or size, or conditions of the environment (e.g., temperature) [19, 20], and the conditions of the surrounding matrix also influence eDNA fate, once released [21].

Persistence of eDNA in sediments has been considered a complicating factor in identifying the recency of occupation by the target organism. Because of the difference in matrix composition (e.g., coarse sand to fine materials, comprising fine sand, silt, or clay particles, as well as organic fractions), longer persistence of eDNA in sediment has been attributed to protection by the formation of biofilms [22, 23]; however, biotic and abiotic factors contribute to DNA degradation over time [24], highlighting that the two contrasting processes (DNA stability vs. degradation) are concurrently occurring. In a closed system, Turner et al. [25], found significantly higher concentrations of bighead carp *(Hypophthalmichthys* sp.) eDNA in sediments than in water, attributing this to the rapid accumulation of high-concentration material (e.g., feces) through settling. Another study found high concentrations of brook trout DNA in stream water samples but no detection in sediment taken from the same locations [26]. Another study of the benthic crested newt (*Triturus cristatus*) found higher concentrations in water, with variable detection rates in multiple sediment compositions [27]. Additional abiotic conditions that affect this difference in persistence include temperature, oxygen, pH, ionic strength, and carbon content [24, 28].

In addition to biological fate, transport of eDNA, particularly in lotic systems, also affects eDNA persistence and detection. Tracking the transport of eDNA in aquatic ecosystems initially relied on models of particle transport in aquatic systems [29], e.g., bacterial transport models [30]. Studies have described detection of eDNA at distances as far as 12.3 km from the source using two common zooplankton species and associated models describe detection much greater (e.g., 50 km); transport of fish eDNA was abundant even at the far distance sampled of 240m in another lotic system [31]. Studies have indicated that distance of detection was species [29] and hydrologically [18] dependent. More recent studies have acknowledged the diversity of DNA sources included in eDNA release (e.g., whole cells, mitochondrial vs nuclear DNA, free (i.e., non-particle bound) DNA, eggs, feces) and the impact on eDNA detection; controlled, *in situ* experiments have been used to measure the components of these processes more comprehensively [31–33]. Improving transport models for eDNA require consideration of the environmental factors that impact transport processes, including deposition, resuspension, degradation [18, 25, 34], and substrate type [35]. Understanding these processes can help to shed more light on the questions often asked as to whether detection of target eDNA represents immediate or distant occupation of the site by the organism, and also whether eDNA can serve as a surrogate for estimating population abundance [36–38].

In this study, we examined the fate and transport of eDNA (i.e., ability to detect target DNA) in water and sediment to identify influences on eDNA in natural system and inform interpretations of positive eDNA results. We developed rates for eDNA shedding and decay in water and sediment using *in vitro* laboratory experiments and then modeled and tested transport of eDNA with in situ experiments in a small stream. Results of this study can help resource managers in interpretation of eDNA results in monitoring programs and direct efforts to eradicate invasive species in aquatic systems. This research advances our understanding of DNA behavior in sediments, which is critical for interpreting eDNA results, refining models, and estimating population abundance of target species.

## Materials and methods

### Laboratory mesocosm

**Fig 1:**
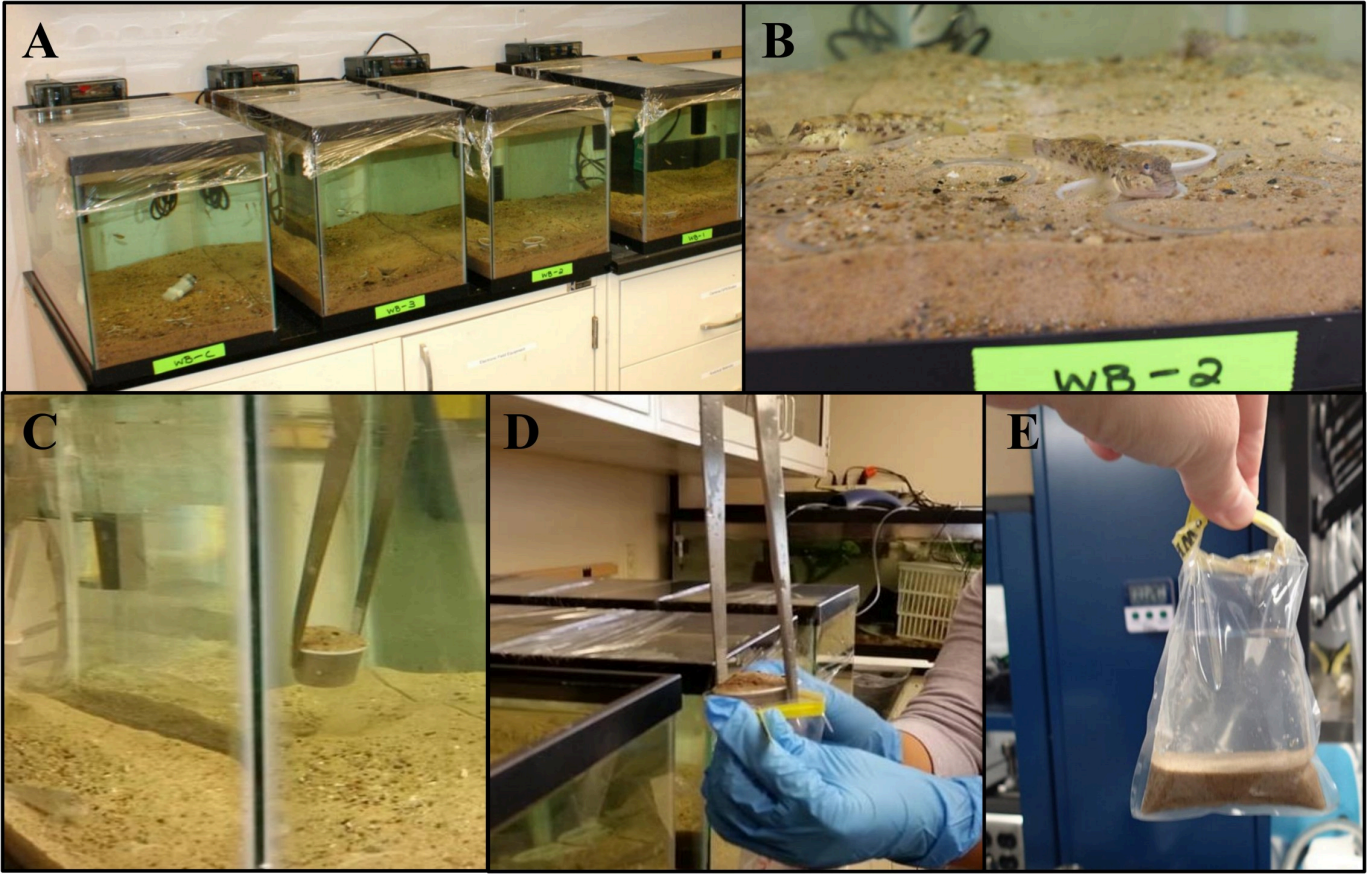
Laboratory mesocosm set-up. (A) Four 10-gal tanks on a lab bench: WB-1, WB-2, and WB-3: three replicate tanks with fish, WB-C: control tank with no fish. All tanks ~1” sediment filled into 48 plastic 1oz cups. HOBO sensor is placed in WB-C for a continuous measurement of temperature and light. All Tanks were wrapped with plastic wrap to reduce evaporation; (B) Round goby in one of the experimental tanks; fish behavior (bottom-dwelling) frequently exposed the sediment to eDNA; (C) Sediment was retrieved in cups using 18” sterilized metal tweezers. Two cups were retrieved from each tank at every sampling interval in random order; (D) Cup and sediment were placed into a Whirl-Pak and placed in cooler; sediment and water stored in separate coolers, transported to USGS laboratory, and processed within 1 hour of collection; (E) 100 mL of sterile PBS was added to the Whirl-Pak containing a cup and sediment; cup was removed using sterile technique. Sediment and PBS were shaken for 2 minutes, and all elutriate was filtered onto a 0.2-micron nitrocellulose filter.

A mesocosm experiment was carried out at the Indiana Dunes National Park laboratory (Chesterton, IN 41.6309°N, -87.0878°W). The bottoms of eight, 10-gallon (38 L) glass tanks were lined with forty-eight, 1 oz (30 mL) disposable plastic cups that were punctured on the sides and bottom to allow for exchange of air and water between sediment in the cups and surrounding area. Each cup-lined tank was filled with one of two custom sand mixtures defined as West Beach ‘WB’ (n = 4) or Portage Lakefront ‘PL’ (n = 4) to a depth of ~2–5 mm above the cup tops (~7.0 kg of sediment per each tank) (see Fig 1A–1E). The two custom sand mixtures were created following Simon et al. [39] to match the particle size distribution of two Indiana Dunes National Park beaches; West Beach (WB; Gary, IN) and Portage Lakefront beach (PL; Portage, IN); these sites were selected because of previous information available on round goby abundance [20] and also because the two represent different sediment composition. Stock sand was collected from local beaches and sieved for 15 mins using a Humboldt motorized sand shaker equipped with following ASTM sieves #: 4, 10, 20, 40, 60, and 100 (See S1 Table) [39]. Stock sand fractions were then used to create custom sand compositions for the mesocosm experiment consistent with ambient field conditions. Target compositions were taken from Simon et al. [39]; generally, PL was more coarse in composition than WB (see S1 Table).

Once sand was added, each tank was filled with tap water (originating from a well source) and labeled. Tanks were outfitted with Aqua Clear 20 hang-on-back filters containing zeolite media and standard floss filters, wrapped with cellophane plastic to minimize evaporation, and left to cure for 24 hours. Cured tanks were then treated with biologically active water from a laboratory stock fish tank, verified free of round goby DNA, and left to further cure for one month. This step was necessary to ensure systems were biologically active and capable of sustaining the round goby for the duration of the experiment.

Wild caught round goby were held in the in a 150 gal (568 L) laboratory holding tank until acclimated to room temperature water and transitioned to daily feeding of commercial pellet food. Acclimated individuals selected for experiments were weighed (4.9–17.1g), sexed (15 female, 15 male), and placed inside each of the experimental tanks with gloved hands after gently patting the fish dry on a clean towel. Six round goby were placed in each of the 3 PL and 3 WB tanks; one PL and one WB tank held no round goby, serving as a negative control (8 tanks, total). Round goby were kept in tanks for 14 days (shedding period); water and sediment samples were collected on 5 separate occasions in duplicate on D1, D2, D3, D7, and D14. Seven tanks (one WB experimental tank was abandoned after a few days due to high fish mortality) were sampled in random order on each sampling occasion to avoid bias and to detect potential cross-tank contamination. Water was collected by twice submerging a sterile 50 mL pipette in the middle of the tank then dispensing the water into Whirl-Pak bags for duplicate samples; additional 70 mL of water per tank was collected for chemical analysis (turbidity, pH, conductivity). Sediment cups were randomly selected and retrieved from the bottom by lifting the 1oz cups using 18-inch long bleach-sterilized, stainless-steel forceps (Fig 1); each cup was lifted slowly allowing excess water in the cup to gently pour back into the tank before placing the entire cup in a Whirl-Pak. Pipettes and forceps were changed for each tank to avoid cross-contamination. After each sampling, 170 mL of autoclaved reverse-osmosis (RO) water was poured into each tank to keep the volume constant; tanks were wrapped with plastic throughout the experiment to minimize evaporation. After sampling on Day 14, round goby were removed from experimental tanks using bleach-sterilized fish nets (separate net for each tank); subsequent water and sediment collections were carried out for 150 days or until no round goby eDNA was detected in a substrate (decay period).

Mesocosm experiments were carried out under a plan approved by the Institutional Animal Care and Use Committee (IACUC) of the National Park Service, Project MWR_INDU_Morris_RoundGoby_2018.A3.

### Field sampling: Nearshore lake

In August 2018 water and corresponding sediment samples were collected in nearshore areas at Portage Lakefront, Portage, IN (41.6268° N, - 87.1772° W) and Washington Park, Michigan City, IN (41.7282° N, -86.9099° W), located along southern Lake Michigan in the Laurentian Great Lakes. Three sites were selected along the breakwall at each location approximately 15–25 meters apart. Triplicate water samples were collected at each site, approximately 20 cm from the lake’s bottom using a modified, 10 ft long telescopic sampling pole affixed with a sterile disposable filtration cup fitted with a 1.5 μm glass-fiber filter (GE Healthcare Life Sciences, Pittsburgh, PA); water was directly filtered and pumped through the tubing attached to a peristaltic pump (Geotech Environmental Equipment, Inc.) into a pre-measured bucket [20]. Filters were placed into sterile 7 mL tubes and transported to the laboratory on ice. After water was collected, triplicate sediment samples were collected by snorkeling. A 1oz plastic cup was pushed into the sandy bottom to collect the top layer (~3 cm) of sediment; the cup was then inverted, slowly lifted, and placed in a Whirl-Pak bag (see Simon et al. 2016 for sediment description). Samples were brought to the surface and stored in a cooler on ice.

### Field sampling: Stream transport

During the summer of 2018, three field experiments were conducted at Brown Ditch (41.6939° N, -86.9483° W), within the Indiana Dunes National Park near Michigan City, IN aimed at exploring eDNA transport using a quantitative polymerase chain reaction (qPCR) method for round goby. Brown Ditch is a small first-order stream that drains wetlands, wooded dune and swale, and residential areas; there is some groundwater input just upstream of the study site. Brown Ditch drains into the larger Kintzele Ditch which outfalls to Lake Michigan. The width of the study area reach was measured as 3.4 m with depth ranging 0.06–0.29 m and discharge calculated as 0.235 cubic feet/second. Prior to field experiments, round goby absence in Brown Ditch was confirmed by field surveys using standard electrofishing methods and eDNA analysis on samples collected. Electrofishing was completed at three locations, one site upstream of the experiment, one within the experimental zone and one downstream of the experiment. Simultaneous collection of water samples (5 1-L samples) for eDNA analysis was conducted upstream of electrofishing locations to confirm absence of round goby.

Field experiments were collected three times in approximately 2-week intervals (7/7/2018, 7/26/2018, 8/8/2018). Two days prior to each experiment, water samples were again collected for eDNA analysis to re-confirm absence of round goby. Sampling stations (6–7) were established at set distances (i.e. 1, 5, 10, 20, 40, 80, 120 m) from the site of planned eDNA introduction. Stream velocity was measured using a fishing bobber that was placed in the stream and timed over a 5 m distance to establish sample collection time intervals (Table 1). For simultaneous sampling, researchers were assigned to each station and remained in the stream with minimal movement to avoid sediment resuspension. A wire cage containing 36, 51, 44 wild caught round goby with cumulative biomasses 403, 406, 405 grams for experiments 1–3, respectively, was deployed upstream of sampling stations. After cage deployment, individual water samples were collected simultaneously at each sampling station at pre-established time intervals. The round goby eDNA cage remained in the stream for 24 hr, and water samples were collected at 2, 4, and 24 hours after simultaneous sampling ended. After 24 hr, the cage (eDNA source) was removed and additional water samples were collected at 48 hours. Field experiments were carried out with approval from the Indiana Department of Natural Resources and the U.S. Department of Interior, National Park Service.

**Table 1:** Timing of sample collection for simultaneous sampling at distances from DNA source (cage containing round goby).

	Elapsed Time (seconds)													
			*Simultaneous sampling*							*Post-Simultaneous sampling*				
Distance (m)	0		12	61	122	243	487	973	1440	7200 (2h)	14400 (4h)	86400 (24h)		172800 (48h)
0	X	DNA source placed	X	X	X	X	X	X	X	X	X	X	DNA source removed	X
1	X		X	X	X	X	X	X	X	X	X	X		X
5	X			X	X	X	X	X	X	X	X	X		X
10	X				X	X	X	X	X	X	X	X		X
20	X					X	X	X	X	X	X	X		X
40	X						X	X	X	X	X	X		X
80	X							X	X	X	X	X		X
120*	X								X	X	X	X		X

Elapsed time was calculated based on stream velocity; sampling at a designated distance was initiated at the time eDNA was anticipated to arrive at that point after DNA source was placed in the stream.

* = samples not collected during Experiment 1

### Sample processing

Water samples collected during the stream transport experiments were pre-filtered through a 1.5 μm glass fiber filter (GE Healthcare Life Sciences, Pittsburgh, Pennsylvania) into a 1 L side-armed flask due to high turbidity and particle content in the stream water. Then, two 300–500 mL aliquots of the pre-filtered water were further filtered through a 0.45 μm nitrocellulose filter (EMD Millipore, Billerica, Massachusetts). The 100 mL (total) water samples collected during mesocosm experiment were filtered through 0.22 μm nitrocellulose filters (EMD Millipore, Billerica, MA).

For sediment samples from the mesocosm study and nearshore lake sampling, 100 mL of sterile phosphate buffer solution (PBS, pH = 7) was poured into the Whirl-Pak; the Whirl-Pak was gently manipulated by hand and the empty cup was removed. The Whirl-Pak was re-tied, and the sand-PBS mixture was shaken for two minutes. The resulting elutriate (entire 100 ml volume) was poured into a sterile funnel, taking care not to introduce sand particles, and filtered through a 0.22 μm nitrocellulose filter (EMD Millipore, Billerica, MA). Because of differences in sediment composition, affecting weight, and differences in volume retrieved, as a result of displacement, eDNA results are presented as copy numbers (CN)/100 ml of elutriate, which assumes complete elutriation of the entire sediment structure collected in each 1-ounce cup.

All filters (water and sediment) from mesocosm and field experiments were placed in PowerWater bead tubes (Qiagen) and stored at -20°C until DNA extraction.

#### eDNA extraction

Filters from all water and sediment samples were extracted using Qiagen PowerWater© kit (Qiagen, Hilden, Germany) according to manufacturer’s instructions with one exception: the final DNA elution step, was performed twice using 50 μL of DNA elution buffer (EB) for a final extraction volume of 100 μL. For stream transport, both 0.45 μm filters were initially processed separately during the lysis steps but loaded into the same spin column resulting in a single DNA extract per sample. DNA concentration for all samples was measured by fluorometric quantification (Qubit® High Sensitivity dsDNA HS Assay, Thermo Fisher Scientific, Waltham, MA), and DNA quality was measured using NanoPhotometer 260/280 ratio (NanoPhotometer Pearl, Implen Inc., Westlake Village, California). DNA extracts were stored at -80°C. Extraction blanks were performed 3 times throughout stream transport and mesocosm extractions and 1 time for nearshore samples; all extraction blanks were free of round goby DNA.

#### Quantitative real-time PCR (qPCR) assays

Samples were analyzed in triplicate (technical replicates) for round goby using the following primer and probe set: GobyCOI-F2*d*: 5’- CTTCTGGCCTCCTCTGGTGTTG-3’, GobyCOI-R2d: 5’-CCCTAGAATTGAGGAAATGCCGG-3’, and GobyCOI-Pr: 5’-6-FAM-CAGGCAACTTGGCACATGCAG-BHQ-3’, slightly modified from Nathan et al. [40]. Modifications included converting degenerate bases to match the nucleotides that were reflected in the aligned round goby sequences [20]. qPCR assays were performed using the Bio-Rad CFX Connect™ Real-time PCR Detection System (Bio-Rad, Hercules, CA) in clear 96 well PCR plates. Protocol followed those described in Nevers et al. [20].

For each assay, amplification efficiency and R^2^ was determined; standard curves for all runs had an R^2^ ≥ 0.99, and amplification efficiency ranged between 89–101%. Quantification of samples was determined from standard curves obtained from six, ten-fold serial dilutions (7.46 X 10^5^–7.46 X 10^0^ CN/ reaction; run in triplicate) of a gBlock® Gene Fragment (Integrated DNA Technologies, Coralville, Iowa), synthesized from a 406 bp DNA fragment. The gBlock® Gene Fragment concentration was determined using a NanoPhotometer Pearl.

Definitions and calculations for the limit of quantification (LOQ) and the limit of detection (LOD) were determined using the discrete threshold approach [41] with a few exceptions. Exceptions include setting different specific criteria levels for LOD probability of detection (92%) and LOQ precision (42%). Eight standard curves (totaling 24 replicates per concentration) were selected from all qPCR runs, which resulted in LOD and LOQ values of 7.46 CN/reaction. Consequently, all starting quantity (SQ) values >7.46 were considered within the range of quantification (ROQ); all SQ values <7.46 were considered as positive, but not quantifiable (DNQ) and were replaced with ½ value of LOQ for statistical purpose (i.e., 3.73) [15]; and all SQ with no exponential curve crossing the threshold value before cycle 40 were considered non-detects (ND) and were replaced with ¼ value of LOQ for statistical purpose (i.e., 1.865).

If the triplicate reactions resulted in an incomplete outcome (ROQ value for 1 or 2 replicates and non-detect for 1 or 2 replicates), the original samples were reanalyzed in triplicate per Goldberg et al. [42] and the average quantities of the second round of amplification were used. All data were averaged across technical and field/mesocosm replicates for use in statistical analysis.

#### Quality assurance

All items used for field sampling were either disposable or bleach-sterilized. Field gear, including coolers, waders/boots, and sampling pole were surface-wiped with 10% bleach wipes (Clorox©, Oakland, CA) and rinsed with reverse osmosis (RO) water using a garden handheld sprayer. All field samples were stored on ice, transported to the laboratory and filtered within 2h. A field blank (i.e., autoclaved RO water inside of the bleach-sterilized polypropylene bottle) was brought to the sampling site each time, each lid was unscrewed on site, exposing the blank to environmental air for 3-seconds; lid was then fastened, and the blank was placed back inside the cooler alongside other samples. Duplicate field samples for the stream transport experiment were collected at random distances and sampling times as quality assurance.

Sampling bottles and items used in laboratory processing were soaked for 10–20 minutes in 30% bleach solution, rinsed thoroughly with autoclaved RO water, and autoclaved at 121°C; tweezers were exposed to UV radiation for 10-minutes instead of autoclaving. To minimize DNA carryover and cross-contamination, nitrile or vinyl gloves were used; gloves were changed after each sample collection between sites and frequently changed in the laboratory. DNA extractions and qPCR assay preparations were performed in biological and laminar flow hoods equipped with UV light; qPCR assay preparations were performed in a dedicated room for preparing reagents for molecular assays.

For the laboratory mesocosm, eight 10-gallon glass tanks and eight Aqua Clear 20 hang-on-back fish filters were soaked for 10–20 minutes in 25% bleach solution and rinsed thoroughly with deionized water. Sterile, disposable 1 oz (~15-mL) plastic cups were used for sediment collection and were removed using bleach cleaned forceps that were long enough to reach the substrate without submerging an entire hand. HOBO temperature loggers (Onset, Bourne, MA) that had been cleaned with bleach wipes and rinsed with autoclaved RO water were attached to bleach cleaned weights and sunk into fixed positions in the tanks to measure light and temperature throughout the experiment. During the field experiment, a Hydrolab MS5.0 sonde (Hydrolab, Loveland, CO) was cleaned and calibrated before being deployed to continuously monitor water quality parameters.

#### Statistical analysis

Statistical analyses and graphical representations were performed using Systat version 13 [43], SPSS version 26 [44], R version 4.0.1 [45], and R tidyverse package [46]. Pearson correlation analysis was used to determine significant relationships between eDNA concentrations in sediment and water, and independent samples t-test, paired t-test, and analysis of variance (ANOVA) were used to determine significant differences in eDNA concentrations between water and sediment, sediment composition type, and sites. Mixed-effects models, with tanks as a random effect and using the lme4 package [47] in R, were used to determine if eDNA (CN/100 mL) differed in water vs. sediment and across PL vs. WB mesocosms. The natural log of CN was used in all mixed-effects models; however, anti-log values are reported for consistency. Significance was tested with a Likelihood Ratio Test using the ANOVA function in R. The Likelihood Ratio Test compares the goodness of fit of two models, the null model (without fixed effect) and the full model (with fixed effect). Ranges and means for CN/1000 mL and CN/100 mL are averaged across field/mesocosm and/or technical (qPCR wells) replicates.

*Decay rates*. Round goby eDNA decay rates were calculated for sediment using a first-order decay model for each mesocosm (PL and WB). An exponential decay rate was calculated following previous decay models [29, 33]:

$$\ln\left(\frac{C}{C_{0}}\right)=-kt$$ (1)

Allowing the model to estimate decay rate and the initial concentration, we use the equation:

$$\ln\left(C\right)=\ln\left(C_{0}\right)-kt$$ (2)

Where C = the concentration of eDNA in CN/100 mL, C_0_ is the initial eDNA concentration, t = time since the start of experiment in hours, k = first order decay rate constant.

A mixed effects model that includes tank as a random factor was used to calculate k and C_0_ using the lme4 package in R. The resulting slope of the line and intercept provided the first-order decay function, k, and the initial eDNA concentration, C_0_, respectively.

Half-life for eDNA persistence was calculated by incorporating C_t_ = C_0_ into Eq (2):

$$t_{\frac{1}{2}}={log}_{1-k}\frac{1}{2}$$ (3)

*Shedding rates*. In order to calculate shedding rate, eDNA concentration must reach steady state: when eDNA shedding is in equilibrium with eDNA decay. Shedding was calculated after eDNA concentration in water and sediment had reached steady state; because not all replicate tanks reached steady state, averages were based only on those that achieved this status. All shedding rates were calculated as net shedding, which incorporated eDNA loss to decay, using k as described above.

$$V\frac{dC}{dt}=S-kCV$$ (4)

Where V is the volume of the tanks in 100mL units, C is the eDNA concentration (CN/100 mL), t is time (hrs), S is the eDNA shedding rate (CN/hr), and k is the calculated first order decay rate constant/hr. At steady state, $\frac{dC}{dt}=0\;and\;S=kCV$. Shedding rates were also calculated per g of round goby (CN/hr/g-goby).

*Brown Ditch transport model*. A simplified, one-dimensional plug-flow reactor model was developed to model round goby eDNA as a function of distance from round goby cage following [33, 48].

$$\frac{\partial C}{\partial t}+u\frac{\partial C}{\partial x}=-kC$$ (5)

where C represents the eDNA concentration (CN/mL), t represents time in hours, μ is the water velocity (m/h) in the streamwise direction (x-direction), and k is the first-order decay constant [20]. For model application, we assumed steady state and solved for Eq 1

$$C=C_{cage}\;e^{-kx/u}$$ (6)

Where C_cage_ represents the concentration of round goby eDNA being shed from the round goby cage and was determined as

$$C_{cage}=\bar{S}\times M\times t$$ (7)

where $\bar{S}$ is the mean round goby eDNA shedding rate in CN/hour/goby, M is the number of round goby/m^3^ inside the round goby cage, and t is steady state time in hours [20].

## Results

### Laboratory mesocosm

**Fig 2:**
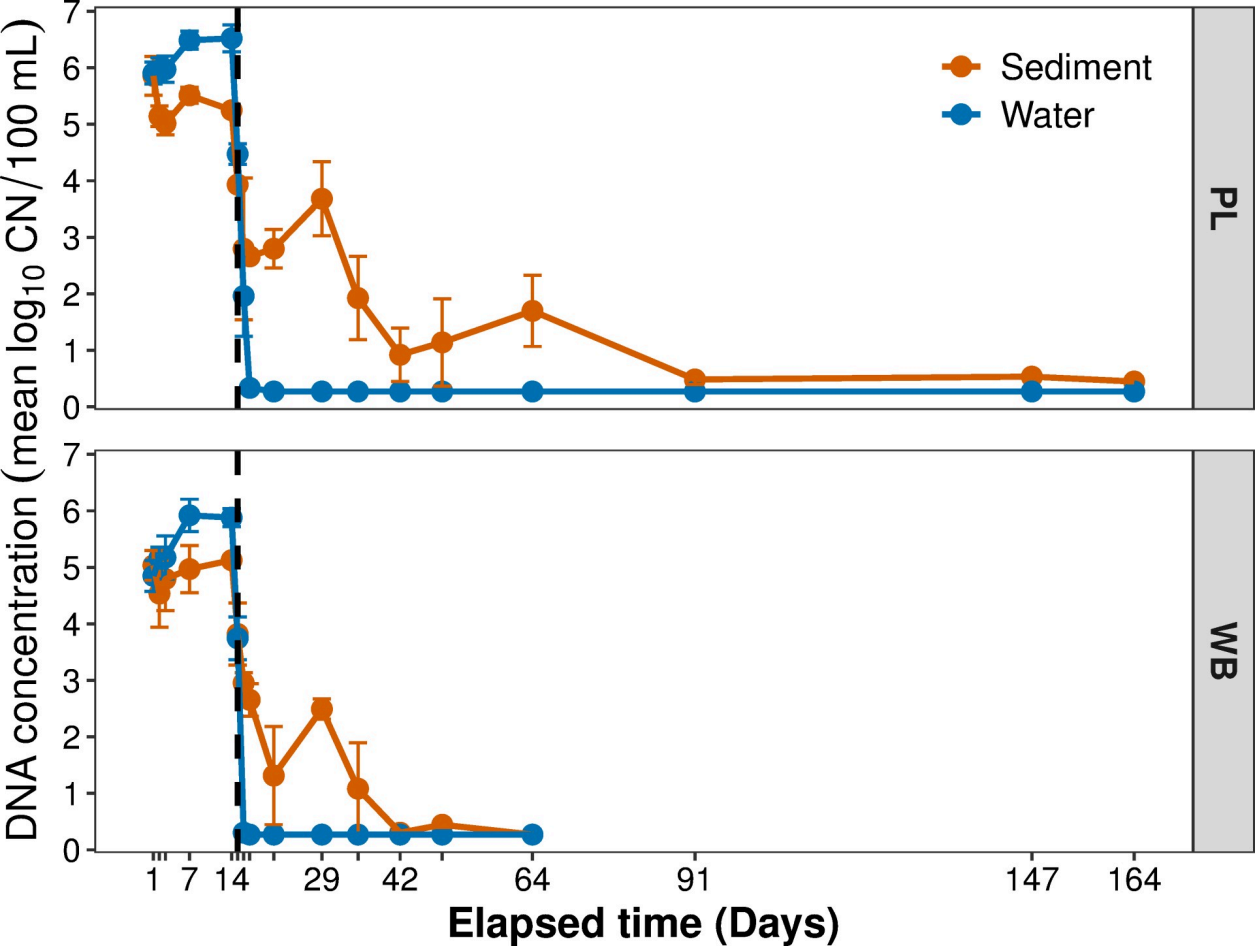
DNA concentration and standard error in water and sediment over time in two experimental mesocosms. Concentration measured in 100 mL of water or 100 mL of elutriate from whole sediment sample. Dashed vertical line indicates when fish were removed from the experimental tanks. PL = Portage Lakefront; WB = West Beach.

Twenty-four hours (24h = D1 sampling time) after round goby were introduced, eDNA copy numbers (CN) in water reached concentrations ranging from 4.849 ± 0.276 to 5.906 ± 0.193 (log_10_ CN/100 mL ± SE) in WB and PL tanks, respectively. At the same sampling time (D1), eDNA concentrations in sediment samples ranged from 5.035 ± 0.263 (WB) to 5.857 ± 0.343 (PL) (Fig 2). Due to high mortality in one WB tank, sampling was abandoned for that tank during the experiment.

#### Water vs. sediment

eDNA concentrations were significantly higher in PL than WB for both water and sediment (F = 16.483, P<0.001 and F = 6.062, P = 0.022, respectively), so further comparisons were done separately for the two sediment types (PL and WB).

Throughout the 14-day shedding period, eDNA CN were significantly higher in water than in sediments for PL and WB tanks (t = -5.249, P<0.001 and t = -2.197, P = 0.041, respectively). eDNA (CN/100mL) in water and sediment were significantly correlated for both PL and WB (Pearson R = 0.806, P<0.001 and R = 0.881, P<0.001, respectively) over the experiment, but when shedding and decay periods were examined separately, water and sediment were correlated only during the decay period for PL and WB (R = 0.372, P = 0.04 and R = 0.584, P = 0.011, respectively) and not during shedding.

In PL tanks, CN in water were significantly higher over time than in sediment according to a likelihood ratio test (χ^2^ (1) = 22.415, p = 2.26x10^-06^); mixed effects model shows water had approximately 6.53 ± 1.39 (model coefficient) more CN than sediment. In WB tanks, CN in water were also significantly higher over time than in sediment according to a likelihood ratio test (χ^2^ (1) = 10.549, p = 0.001163); mixed effects model shows water had approximately 3.21 ± 1.39 (model coefficient) more eDNA than sediment.

After fish were removed, round goby eDNA concentrations declined rapidly in water; overall non-detects were recorded at D17 (WB) and D21 (PL). eDNA concentrations in sediments declined more gradually and were variable between replicated tank systems; overall non-detects were recorded in WB tank at D64 (50 days after fish removal), but eDNA was still recorded in some subsamples in PL tank at D164 (150 days after fish removal). Nonetheless, eDNA concentrations in sediment were patchy, resulting in inconsistencies of non-detects among replicates. During this decay period, eDNA concentrations were significantly higher in sediment samples than in water (F = 23.396, P<0.001), but they were not significantly different between WB and PL tanks.

#### Influence of sediment type (PL vs. WB)

Highest eDNA CN in water samples were recorded on D14 for PL (6.521 ± 0.238) and on D7 for WB (5.92 ± 0.285). Highest eDNA CN in sediments were recorded on D1 in PL (5.857 ± 0.343) and D14 in WB (5.127 ± 0.028).

In WB tanks, CN in water were significantly lower than in PL over time according to a likelihood ratio test (χ^2^ (1) = 34.685, p = 3.9x10^-09^); the mixed effects model shows WB had approximately 0.12 ± 1.26 (model coefficient) less eDNA than mesocosm PL. Likewise, CN in sediment in WB had significantly lower eDNA than PL according to a likelihood ratio test (χ^2^ (1) = 5.7307, p = 0.01667); the mixed effects model shows WB had approximately 0.41 ± 1.46 (model coefficient) less than mesocosm PL. During the decay period, there was no significant difference between eDNA concentrations in PL and WB tanks for either water or sediment.

#### Decay rates

First order decay constants were calculated as k = 0.002442 (±0.000557 SE) in PL sediment and k = 0.006259 (±0.001165 SE) in WB sediment (Table 2). Sediment eDNA in WB was non-detectable using our qPCR assay at 50 days in one tank and at 21 days in the other; in the PL tanks, eDNA was detected, albeit in very low numbers, at the conclusion of the experiment. First order decay constants were not obtained for water in PL and WB due to low CN variability, as a result of rapid decline to non-detect during the decay period. Half-life estimations for eDNA were shorter for WB sediment: estimates were 110 hours for WB and 283 hours for PL.

**Table 2:** Shedding and decay constants.

Rate	Water-PL	Water-WB	Sediment-PL	Sediment-WB
Decay constant, k (± SE)	NA	NA	0.002442 (0.000557)	0.006259 (0.001165)
Shedding/Accumulation (CN/hr)	2.7x10^7^	1.1x10^7^	1.5x10^5^	NA
Shedding/Accumulation (CN/hr/g-goby)	5.7x10^5^	2.3x10^5^	2.7x10^3^	NA

Calculated decay constants (k) and shedding (water)/accumulation (sediment) rates calculated from mesocosm experiments using sediments from Portage Lakefront (PL) and West Beach (WB) sites. Shedding/accumulation is calculated as copy numbers (CN)/hour (hr) and also as CN/hr/gram of round goby (g-goby). NA = rates could not be calculated due to lack of stable steady-state conditions.

#### Shedding rates

Steady state was reached in water after days 3 (PL) and 7 (WB) in one replicate tank; steady state was reached in the sediment after day 3 (PL) in one replicate tank. Steady state was not attained in the other tanks, so calculations are based only on those that achieved steady state. Because no decay rate was obtained for water in the mesocosm experiments outlined here, previously published decay rates for water were used to calculate shedding [20]. A mean shedding rate for water was 2.7x10^7^ CN/hr or 5.7x10^5^ CN/hr/g-goby (PL); and 1.1x10^7^ CN/hr or 2.3x10^5^ CN/hr/g-goby (WB) (Table 2). A mean accumulation rate for PL sediment was 1.5x10^5^ CN/hr or 2.7x10^3^ CN/hr/g-goby.

### Nearshore lake

Round goby eDNA was consistently detected in Lake Michigan nearshore water and sediment samples at two breakwalls. eDNA in water varied from 0.94 ± 0.604 to 2.18 ± 0.155 (log_10_ CN/100 mL ± SE) and in sediments from 0.27 ± 0.0 to 3.78 ± 0.093 (log_10_ CN/100 mL ± SE). The correlation between CN in water and sediments was fairly strong but not statistically significant (Pearson R = 0.731, P = 0.099); CN in sediments was higher than in water samples, 2.874 ± 0.568 and 2.071 ± 0.313 respectively, but the difference was not significant (t = -1.379, P = 0.198).

### Stream transport

**Fig 3:**
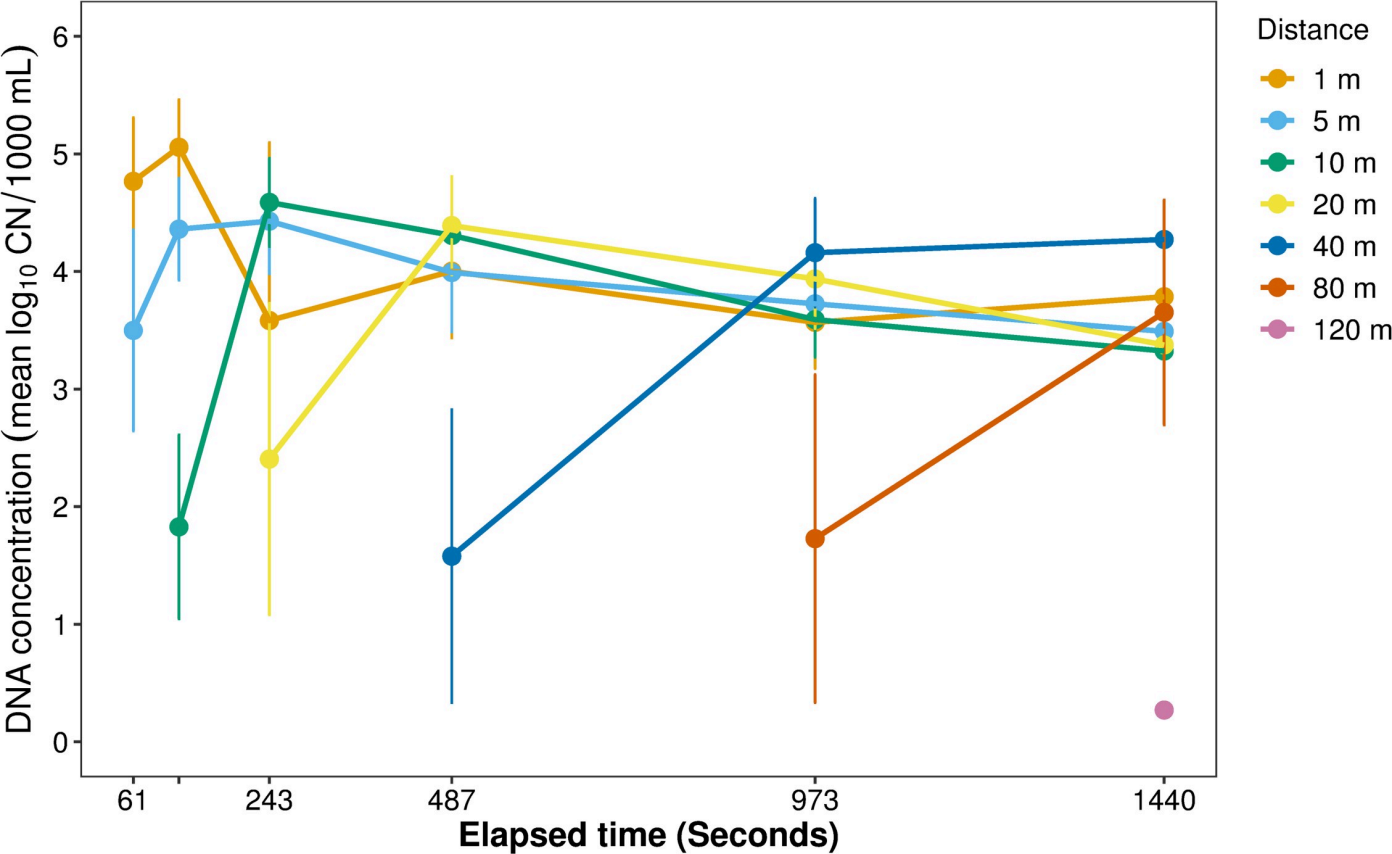
Concentrations of eDNA (log_10_ CN/L) over time during simultaneous sampling event. Samples were collected simultaneously at each distance from the DNA source (cage containing round goby), with first sample collection at a given distance initiated by calculated estimate of DNA arrival at the site. Rising arm at each location indicates first sample at each distance was collected during increasing phase. Once eDNA had saturated the stream, there was no significant difference in concentration between sampling distances.

**Fig 4:**
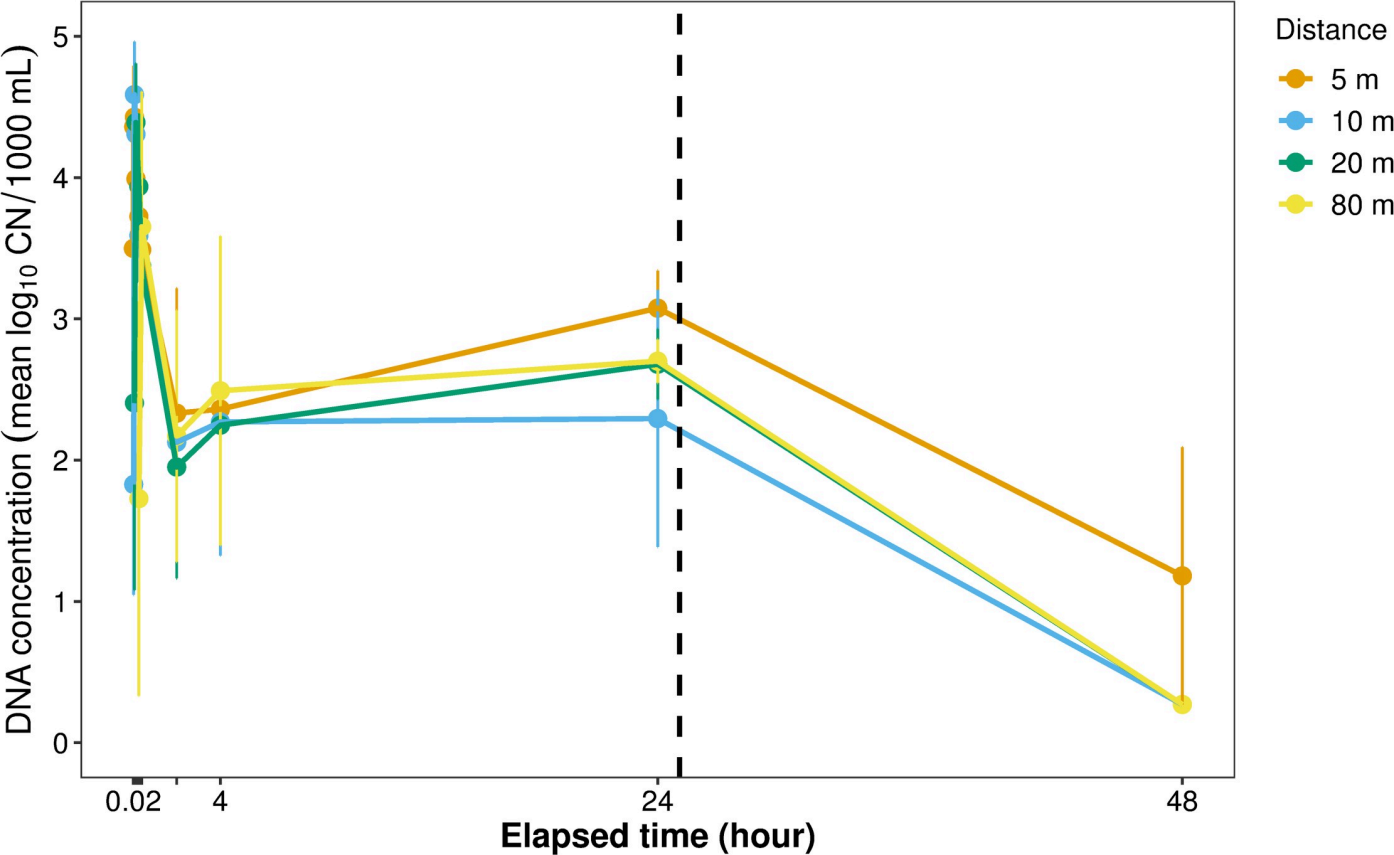
Concentration of eDNA over simultaneous sampling and after round goby removal. Concentration of eDNA (log_10_ CN/L) and standard deviation over time at four distances (5, 10, 20, 80 m) from DNA source (cage containing round goby) placed in stream. Samples were collected at 6 times within the first 24 minutes of cage placement (see Fig 3), then at 2, 4, 24, and 48 hours. Dashed vertical line indicates time of cage/DNA source removal. There was no significant difference in concentration between sampling distance, indicating steady state along the entire distance measured.

During the intensive sampling (TR) phase of the stream experiment, round goby eDNA was consistently detected at all seven stations with lowest eDNA concentration at 120 m (0.271 ± 0.0 log_10_ CN/1000 mL ± SE), which was furthest downstream, and highest at 1 m (5.057 ± 0.404), which was closest to the eDNA source (round goby cage). While CN at 1 m distance ranged widely from 1.309 ± 0.93 to 5.057 ± 0.404 over the seven samplings, concentrations were more consistent at 5 m (3.490 ± 0.19 to 4.428 ± 0.447) and 20 m (2.405 ± 1.324 to 4.39 ± 0.418) distances. Simultaneous sampling showed a clear rise in eDNA concentration at each site with initiation of sample collection (Fig 3). After the simultaneous sampling was completed, CN leveled off, and there was no significant difference in variation among sample times (2, 4, 24 hr post-removal) (F = 0.339, P = 0.714) (Fig 4). After the eDNA source (i.e., experimental cage) was removed from the stream, the signal decreased rapidly, with CN at 48 hr significantly lower than at 24 hr (t = -10.202, P<0.001).

#### Brown Ditch eDNA transport model

**Fig 5:**
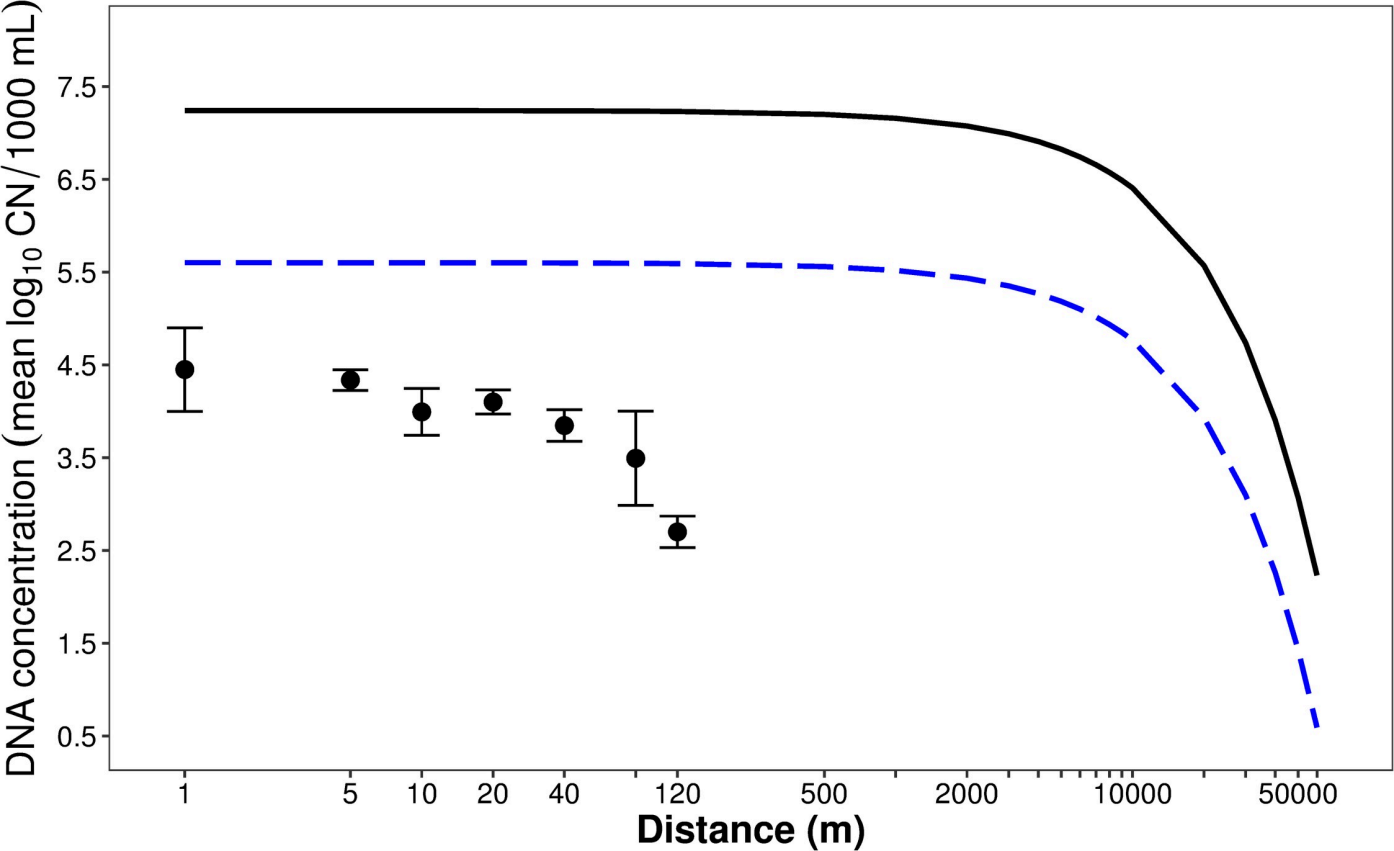
Model of eDNA transport in a small stream. Solid line is model result using a DNA source of average 44 round gobies, shedding and decay rates calculated through mesocosm experiments, and measured stream flow and depth. Points (with standard error) are measured concentrations of eDNA over the course of the experiment. Blue dashed line indicates modified transport curve, with number of round gobies scaled back to one individual.

Round goby eDNA concentration (C_0_ = C_cage_) was calculated as 17,457 CN/mL based on the following: Brown Ditch average depth was 0.11 m; round goby density was 96 round goby/m^2^, steady state was 6 h, and the shedding rate was 3,378,574 CN/hour/goby (calculated using [20]). Along with the calculated C_cage_, a 302 m/hour velocity, the decay constant (k = 0.058) [20], and the limit of quantification of 0.456 CN/mL (based on 1000 mL of Brown Ditch water filtered), the model predicted that round goby eDNA could be detected as far as 54.89 km downstream of the eDNA source (Fig 5). We then extended the model calculation to the same distances (1–120 m) in which water was collected during the Brown Ditch transport experiment. Model estimated eDNA (log_10_ CN/1000 mL) were then compared to those of the qPCR results obtained from water collected over the first 24-hour period. The model estimate was generally three log higher than our qPCR results, and spatial decline was marginal over the sampled distances (1–120 m). However, estimated eDNA declined more rapidly when reaching distances closer to the predicted downstream value (54.89 km or 54890 m).

## Discussion

DNA is acutely responsive to the biological, chemical, and physical conditions of the environment into which it is released, whether the eDNA is present in intact cells, free floating, or particle bound. Presenting eDNA as a potential management tool requires a comprehensive understanding of the impact of these processes on eDNA released by biotic communities under ambient conditions [18, 49]. A fully integrated model will require consideration of all conditions, but quantifying individual impacts (e.g., shedding and decay rates, transport) can be done experimentally and systematically. In this study, we isolated sediment as a factor to determine its influence on eDNA stability, long term persistence, and environmental degradation and then attempted to integrate this into a stream transport model, using invasive round goby as a model fish or eDNA source.

### Influence of sediment on estimates of eDNA

The presence of sediment influenced estimates of eDNA shedding and decay because it provides a potential sink for accumulation. Several studies have investigated differences in eDNA degradation in water vs. sediment with widely variable results. Turner et al. [25] and Honjo et al [50] both found significantly higher concentrations of eDNA in sediments than in water, but Baldigo et al. [26] instead found that water was more effective at retaining brook trout eDNA than sediment. Different conclusions may be the result of where eDNA is present in the system, whether particle-bound or freely suspended, which can affect relative eDNA concentration in water and sediment. Findings presented here show a slower accumulation but longer retention of round goby eDNA in tank sediments. Overlying water instead showed a high spike in eDNA when fish were added to the system and a rapid decline when the fish were removed. Similarly, our stream transport experiment showed no accumulation of eDNA in sediment except for one instance of detection at the 1-m site closest to the eDNA source (round goby cage) and only after 24 hrs of exposure [51], indicating potentially slow accumulation. Stoeckle et al. [52] showed that the presence of sediment alone contributed to lower eDNA detection in water, further supporting the concept of gradual accumulation in the system as a result of sediment accumulation. This finding could guide the interpretation of eDNA detection in the two matrices, with eDNA in water representing more recent, immediate presence of an organism and eDNA in sediment eDNA representing longer term persistence or long-term occupation by an organism. The findings are similar to research in shoreline environments that identifies DNA in these two matrices as a means of monitoring two different populations of microbes [53].

The amount of time that sediment is exposed to an eDNA source influences the overall accumulation, as particle-bound eDNA, especially DNA fragments attached to finer particles (e.g., clays, organics, colloidal fraction and lighter cellular debris) takes longer to settle in lentic habitats. While eDNA could be detected in our mesocosm experiments in both water and sediment after 24 hours, eDNA was consistently higher in water than in sediment during the time round goby were present. In the larger stream system, 24 hours was not enough time for eDNA to accumulate in sediment (see above, data not shown) except in very close proximity to the eDNA source. We tested this relationship in Lake Michigan at locations that had large well-established populations of round goby, and there was no significant difference in eDNA concentrations between water and sediment. Given enough time, there appears to be a gradual accumulation of eDNA in sediment, and at some point, a steady state or equilibrium appears to be reached.

Subsequent to accumulation, the rate of decay within the sediment vs. water also influences estimates of eDNA concentration. In our experiments, decay rates in sediment were calculated as k = 0.002–0.006, which is an order of magnitude lower than decay rates previously measured under similar circumstances in water: k = 0.043–0.058 [20]. There are fewer studies measuring eDNA decay or degradation in sediment, but Sakata et al. [54] also found lower rates of decay in sediment than in water, with rates in sediment even a magnitude lower than our findings (k = 0.0003) for two fish species. Interactive factors that affect rates have been identified, including biochemical oxygen demand and initial concentration of eDNA present [21] and bacterial activity [55], as well as protection from other adverse conditions (e.g., enzymatic degradation by binding to clay or organic fractions) [22, 56]. Sediment has been long recognized for its protective characteristics that extend survival of microbes [22, 53]. Results presented here support this concept for eDNA, where sediment creates an environment representing a more stable, long-term pool of eDNA [20, 54, 55]. Indeed, Turner et al. [25] estimated persistence of eDNA in sediments for up to 3 months, and eDNA extracted from deep sediment cores has been used for analyzing centuries-old eDNA in studies of ancient ecosystems [13].

The dual actions of accumulation and decay or degradation are mediated by multiple factors together and individually, including sediment composition. Sediment size and composition has been explored as a factor in eDNA retention. Both Jerde et al. [32] and Aubenau et al. [57] determined that finer particle (e.g., clays and organic fractions) sizes were more effective at retaining eDNA, something Aubenau attributed to higher conductivity among particles. Mesocosm experiments presented here showed similar results, with significantly higher eDNA accumulation in sediments of PL, which was composed of finer sediments than WB [39]. Fewer studies have examined the impact of sediment size on eDNA decay, but in a controlled experiment, Buxton et al. [27] found a difference in detection rates among sediment types only between control and topsoil sediment, a difference they attribute to PCR inhibition. In this study, sediment size influenced eDNA decay, with lower rates of decay in finer particles. This, too, was reflected in our experiments, with a lower decay rate calculated for PL.

### Transport of eDNA in a small stream

Early detection of an invasive species is one of the widely touted applications of eDNA, but it is critical that detection lead to mitigation to prevent spread; this requires an estimation of how far the eDNA may have traveled from the source organism(s). Using a small, remote stream, we were able to perform a controlled experiment to estimate eDNA transport. The initial simultaneous sampling at multiple distances from the eDNA source showed remarkably consistent concentrations of eDNA—with no significant difference by distance—once the stream was saturated with the source, also seen by Jane et al. [31], and we were able to catch the rising arm of the curve to steady state (Fig 3).

The actual amount of eDNA detected in the stream and thus the starting point for modeling transport was far lower than anticipated based on our mesocosm calculations; Nukazawa et al [48] also predicted a higher eDNA shedding—10X higher—than what they observed in the field. Multiple factors could have influenced this result. Notably, water temperature in the stream was variable and reached 28°C during one of the experiments (see S2 Table), which is far higher than the controlled 19°C conditions under which shedding rate was calculated [20]. While temperature generally does not have a significant impact on shedding rates [15, 58], it is possible that the transition from indoor tanks to a natural stream with diurnal fluctuations could have affected the gobies’ shedding rate. Further, our model used a shedding rate for water derived from a previous study [20] that did not include sediment in the experimental setup; as mentioned previously, the presence of sediment alone impacts amount of eDNA detected in the water [52] and could therefore affect estimates of shedding/available eDNA for the model.

Estimated distance that eDNA could travel (54.89 km) was consistent with some published model estimates [29] but far greater than reported by others [33], a factor that is largely affected by stream velocity and complexity. Our field sampling validation exhibited a curve in eDNA decline over distance to that modeled, but with a lower initial concentration of available (shed) eDNA and perhaps a more rapid decline. Because the model incorporates only shedding rate, decay rate, depth, and velocity, the physical intricacies of a stream are not included. This would include meanders, variation in substrate types [57], depth changes, and slope [59], all of which may affect both the initial eDNA concentration and the rate of decline. Further experiments using different fish species and water bodies could be used to validate and refine transport models. In this study, while we had hoped to integrate the impact of sediment on transport, we only collected 6 sediment samples that had any detectable eDNA present, so interactive effects could not be tested.

## Conclusions

The use of eDNA for early detection of invasive species and for low density species continues to become a reality as the intricacies of its behavior are described and more informed interpretations of results can be made. In lotic systems, the transport of eDNA from its source is almost inevitable once it is released into the water, and the results presented here, showing consistency in concentration at sites many meters from release, indicate that with enough eDNA present, monitoring can be limited to representative sites. Results also showed that shedding in the field may be far lower than what is measured in a laboratory setting, but if this is consistent among studies, it can potentially be accounted for in model development; regardless, the pattern of decline was like that modeled. Finally, while the development of models for biological, chemical, and physical factors affecting eDNA concentration are providing great strides in understanding eDNA in the environment, there is evidence that many of these interact, and thus a comprehensive model would be needed to consider cumulative and interactive factors. Together, this information may be able to help scientists and managers determine monitoring and mitigation strategies that maximize resources to provide interpretable and rapidly available results.

## Supporting information

S1 Table Summary temperature data. Data were collected during stream transport experiments using a continuous monitoring multiprobe instrument.
(XLSX)

S2 Table Sediment composition. Targeted sediment composition is the individual weight percent finer than each US Standard Sieve size. Percent composition from Simon et al. [39].
(XLSX)
